# Frailty in Older Patients with Transthyretin Cardiac Amyloidosis

**DOI:** 10.3390/jcm12247507

**Published:** 2023-12-05

**Authors:** Stéphanie Cazalbou, Louise Naccache, Sandrine Sourdet, Eve Cariou, Pauline Fournier, Fati Nourhashemi, Laurent Balardy, Olivier Toulza, Olivier Lairez, Zara Steinmeyer, on behalf of the Toulouse Amyloidosis Research Network

**Affiliations:** 1 Department of Cardiology, University Hospital of Toulouse, 31400 Toulouse, France; cazalbou.s@chu-toulouse.fr (S.C.); cariou.e@chu-toulouse.fr (E.C.); fournier.p@chu-toilouse.fr (P.F.); 2 Cardiac Imaging Center, University Hospital of Toulouse, 31059 Toulouse, France; 3 Geriatrics Department, Internal Medicine and Cardiogeriatry Unit, Toulouse University Hospital, 31059 Toulouse, France; naccache.l@chu-toulouse.fr (L.N.); sourdet.s@chu-toulouse.fr (S.S.); nourhashemi.f@chu-toulouse.fr (F.N.); balardy.l@chu-toulouse.fr (L.B.); toulza.o@chu-toulouse.fr (O.T.); steinmeyer.z@chu-toulouse.fr (Z.S.); 4 French National Institute of Health and Medical Research, Toulouse III Paul Sabatier University, 31062 Toulouse, France; 5 Medical School of Medicine, Toulouse III Paul Sabatier University, 31062 Toulouse, France; 6 Department of Nuclear Medicine, University Hospital of Toulouse, 31059 Toulouse, France

**Keywords:** ATTR cardiac amyloidosis, frailty, G8 score

## Abstract

Background—Transthyretin cardiac amyloidosis (ATTR-CA) prevalence increases with age. The interplay between frailty and heart failure has been increasingly recognized. The objective of this study is to compare clinical, biological, and transthoracic echocardiography (TTE) characteristics of older ATTR-CA patients according to the G8 frailty screening tool. Methods—Patients over 75 years old with a confirmed diagnosis of ATTR-CA were included between January 2020 and April 2021. All patients underwent a routine blood test, TTE, and a functional assessment with a six-minute walking distance test (6MWD) or cardiopulmonary exercise testing (CPET), and the G8 score was calculated. Results—Fifty-two patients were included. Thirty-nine (75%) patients were frail and their mean NYHA stage was more severe (2.2 vs. 1.7; *p* = 0.004); 62% of them had a Gilmore stage of 2 or 3 (*p* = 0.05). Global left ventricular strain (GLS) was lower (−11.7% vs. −14.9%; *p* = 0.014) and the interventricular septum was thicker (18 ± 2 mm vs. 17 ± 2 mm; *p* = 0.033) in frail patients. There were no significant differences according to functional tests. Conclusion—The majority of older patients with ATTR-CA are frail according to the G8 score. They are more symptomatic and have an increased cardiac involvement and a poorer prognosis, requiring more personalized cardiac management.

## 1. Introduction

Transthyretin amyloidosis is a disease caused by transthyretin misfolding, resulting in the formation of amyloid fibrils that aggregate in certain organs, including the heart. Although diagnosis theoretically requires histological evidence of amyloid deposits within the organ, the recent development of bone scintigraphy as a non-invasive test for the diagnosis of transthyretin cardiac amyloidosis (ATTR-CA) in the absence of monoclonal gammopathy has enabled the review of the epidemiology of this disease, which proves to be more frequent than historical data might have initially suggested. There are two types of transthyretin amyloidosis: wild-type transthyretin amyloidosis (ATTRwt) and variant ATTR (ATTRv). ATTRwt mainly affects older men, and certain autopsy studies have shown that 25% of patients aged above 80 years old have transthyretin amyloid myocardial deposits [1,2]. However, the presence of amyloid deposits does not necessarily indicate underlying pathology, and most of these patients were never diagnosed with amyloidosis in their lives. On the other hand, in the field of heart diseases, ATTR-CA is thought to affect 13% of patients over 60 years with heart failure and preserved ejection fraction (HFpEF) [3] and 16% of patients with severe aortic stenosis undergoing percutaneous aortic valve replacement [4], both conditions associated with aging. ATTR-CA is associated with an increased risk of hospitalization for heart failure, which is accompanied by a deterioration in prognosis, with a median survival between 24 and 69 months according to the Gilmore staging system [5]. Given the increasing age of the population and the prevalence of this disease in the elderly population, ATTR-CA is expected to become a public health issue in the years to come [6].

Given the challenge of meeting the needs of increasingly aging populations with multimorbidity and declining physical function, frailty has become a topic of growing interest in medical research [7]. Frailty is an age-related decline in biological reserves leading to increased vulnerability to adverse health events [8]. Frailty increases with age, affecting around 26% of patients aged above 85 years old, and is accompanied by a risk of increased cardiovascular mortality compared with robust subjects [9]. Frailty is a significant predictor of adverse health outcomes [10], hospitalizations for acute heart failure [11], and more severe prognosis in cardiovascular disease [12,13]. Despite the prevalence of ATTR-CA and frailty in older patients, studies on this subject remain sparse [14,15].

The Comprehensive Geriatric Assessment (CGA) is the best care approach in managing frailty [16]. However, administration of CGA in ambulatory settings is often time consuming due to the use of multiple scores, so assessment of frailty is often difficult [17]. As such, a rapid frailty screening test is necessary for cardiologists when discussing integrative care in patients with ATTR-CA. The G8 score was designed to identify frail older cancer patients who could benefit from a CGA prior to oncological therapy decision-making [18] and has been evaluated in patients with certain cardiac diseases [19]. 

The aim of this study was to describe ATTR-CA characteristics in older patients according to their frailty status using the G8 score.

## 2. Materials and Methods

### 2.1. Population

Patients with confirmed or suspected cardiac amyloidosis referred to the cardiology department of Toulouse University Hospital, which is a reference center for amyloidosis in the Occitanie region in France, for further cardiac assessment between January 2020 and April 2021 were screened for the study.

Inclusion criteria were patients aged above 75 years old with confirmed ATTR-CA. Diagnosis of ATTR-CA was based on positive ^99m^Tc-hydroxymethylene-diphosphonate scintigraphy (^99m^Tc-HMDP) with a Perugini score of 2 or 3, without a monoclonal gammopathy [20]. In the presence of monoclonal gammopathy, the diagnosis was confirmed by histological analysis, either on myocardial biopsy or on extracardiac biopsy in the presence of suspected amyloid cardiomyopathy with a left ventricular septal thickness over 12 mm.

Exclusion criteria were patients with other types of cardiac amyloidosis (typically light-chain amyloidosis), patients who did not wish to undergo further cardiac or geriatric examination, or those who were not in capacity to give their consent to participate.

During a day in hospital, all patients underwent a transthoracic echocardiography (TTE), a six-minute walking test (6MWT) and/or cardiopulmonary exercise testing (CPET), and a blood test with a genetic analysis to distinguish ATTRwt from ATTRv.

### 2.2. Demographic, Medical, and Geriatric Data

Patients’ demographic characteristics (age and sex), clinical data (symptoms, comorbidities, treatments, weight, body mass index (BMI)), and laboratory results (NT-proBNP, creatinine, albumin, high-sensitivity troponin T, hemoglobin) were obtained from electronic medical records during patient ambulatory hospitalization. All medications (cardiovascular and non-cardiovascular) were quantified and then dichotomized to quantify patients receiving more than 3 medications.

A geriatric assessment was performed in all patients, including the G8 score [18,21] to assess patients’ frailty status and the ADL score [22]. The ADL score assesses overall functional activity: bathing, dressing, going to the toilet, transferring, continence, and feeding. The ADL score ranges from 0 to 6, with 0 referring to patients totally dependent for the 6 basic activities of daily living, and 6 to autonomous patients. The G8 score consists of eight questions on food intake over the past 3 months; weight loss during the last 3 months; mobility; neuropsychological problems; body mass index; taking more than three prescription drugs per day; self-rated health status; and age [21]. The total G8 score varies from 0 to 17. A higher score indicates a better health status. A G8 score ≤ 14 indicates that the patient is at risk of frailty and a CGA must be performed for a more complete geriatric assessment by a geriatrician.

According to the G8 score, patients were dichotomized into 2 groups: a group identified as frail, when the G8 score was ≤14, and non-frail, with a G8 score > 14 [18].

### 2.3. Functional Capacity

The New York Heart Association (NYHA) classification was used to measure cardiac functional capacity [23]. Following patients’ physical capacity, they underwent either 6MWT or CPET. For the 6MWT, patients were instructed to walk at a self-selected pace for 6 min. The total distance covered was measured in meters [24]. Patients with a walking distance of less than 300 m were considered to have poor performance [25]. Predicted six-minute walking distance (6MWD) was calculated with the Equation 6MWD = (7.57 × height) − (5.02 × age) − (1.76 × weight) − 309 for men; and 6MWD = (2.11 × height) − (2.29 × weight) − (5.78 × age) + 667 for women [26]. CPET was performed on a bicycle-ergometer at 10 watts/min with workload increments up to exhaustion (peak respiratory exchange ratio > 1.1). The maximal peak oxygen consumption (Peak VO_2_) was calculated according to Wasserman et al. [27]. 

### 2.4. Transthoracic Echocardiography

TTE was performed and analyzed by independent investigators blinded to the clinical characteristics of the patients with either a Vivid E95 or Vivid S70 ultrasound system (GE Healthcare, Chicago, IL, USA) using a 3.5 MHz transducer. Image analyses were performed offline using the EchoPAC V.202 software (GE Healthcare, Chicago, IL, USA). Two-dimensional and Doppler echocardiography measurements and quantification were performed according to the American Society of Echocardiography and the European Association of Cardiovascular Imaging guidelines [28,29]. The following measurements were collected: diastolic parameters, including early (E) and late (A) diastolic mitral inflow velocity peak; E/A ratio; lateral mitral annulus diastolic early peak velocity (Ea); systolic tricuspid annular velocity peak; tricuspid annular plane systolic excursion (TAPSE); and peak tricuspid regurgitation velocity. All Doppler measurements are made over three cardiac cycles and averaged. Left ventricular end-diastolic and -systolic volumes, and ejection fraction are measured using the modified biplane Simpson’s method from apical two- and four-chamber views. Global longitudinal strain (GLS) was calculated from the average of the segmental strain on a 17-segment model, using two-dimensional speckle tracking of greyscale images from the apical four-chamber, two-chamber, and three-chamber views [30,31]. A ‘bull’s-eye’ plot illustrating segmental longitudinal strain was automatically generated. The strain values for the six basal, six mid, and five apical segments of the left ventricle were averaged to obtain three regional longitudinal strain values. The apex-to-base gradient in regional longitudinal strain (relative apical sparing) was calculated using the formula described by Phelan et al.: relative apical longitudinal strain = average apical longitudinal strain/(average basal longitudinal strain + average mid longitudinal strain) [32].

### 2.5. Prognostic Score

The staging prognosis system proposed by Gillmore et al. was calculated for each patient according to N-terminal pro-B-type natriuretic peptide (NT-proBNP) level and estimated glomerular filtration rate (eGFR) according to the standard MDRD formula [5]. Stage I was defined as NT-proBNP ≤ 3000 ng/L and eGFR ≥ 45 mL/min, Stage II as NT-proBNP > 3000 ng/L or eGFR < 45 mL/min, and Stage III was defined as NT-proBNP > 3000 ng/L and eGFR < 45 mL/min. Patients with a higher stage were those with a poorer prognosis.

### 2.6. Statistical Analysis

Continuous variables were tested for normal distribution using the Kolmogorov–Smirnov test and are expressed as mean ± standard deviation. Laboratory findings were not normally distributed and are presented as medians with interquartile ranges (IQR). 

Group comparisons were made using non-parametric Kruskal–Wallis tests or ANOVA for continuous variables and χ^2^ tests for categorical variables, using Bonferroni corrections for multiple comparisons. Differences are considered statistically significant for *p*-values < 0.05. All analyses were performed using standard statistical software SPSS version 20 (SPSS Inc., Chicago, IL, USA).

## 3. Results

### 3.1. Baseline Characteristics and G8 Score

A total of 52 patients were included. Forty-six (92%) patients had ATTRwt, four (8%) had ATTRv, and two patients did not undergo genetic testing. The mean age was 84 ± 4 years and 42 (80%) patients were male. Forty-eight (92%) patients are treated with tafamidis. Patients were mostly independent with an average ADL score of 5.5 ± 1.0; only two (4%) patients had an ADL score < 3. 

Table 1 presents demographic and medical characteristics according to the frailty status.

The two groups, frail and non-frail, were comparable according to cardiovascular comorbidities and medication upon admission. Frail patients were older and more dependent with a lower ADL score than non-frail patients. Frail patients were more symptomatic with a greater NYHA stage and had a trend of higher NT-pro-BNP and high-sensitivity troponin T levels.

### 3.2. Functional Capacities

Forty-seven (90%) and twenty-nine (56%) patients underwent 6MWT and CPET, respectively. The limited number of patients who performed CPET was due to limited mobility not allowing them to perform the test. There was no significant difference between the two groups according to the patients’ 6MWD and peak VO_2_ (Table 2). The mean NYHA stage was higher in frail patients.

### 3.3. Transthoracic Echocardiographic Parameters

There were no significative differences according to the G8 score in left ventricular ejection fraction and diastolic function, or right ventricular systolic function.

There was a decrease in the GLS in the frail group, with a value of −11.7% and −14.9% in frail and non-frail patients, respectively (*p* = 0.014). Frail patients had more pronounced left ventricular wall thickness with a thicker interventricular septum than non-frail patients.

Echocardiographic parameters are presented in Table 3.

### 3.4. Prognostic Score

**Figure 1:**
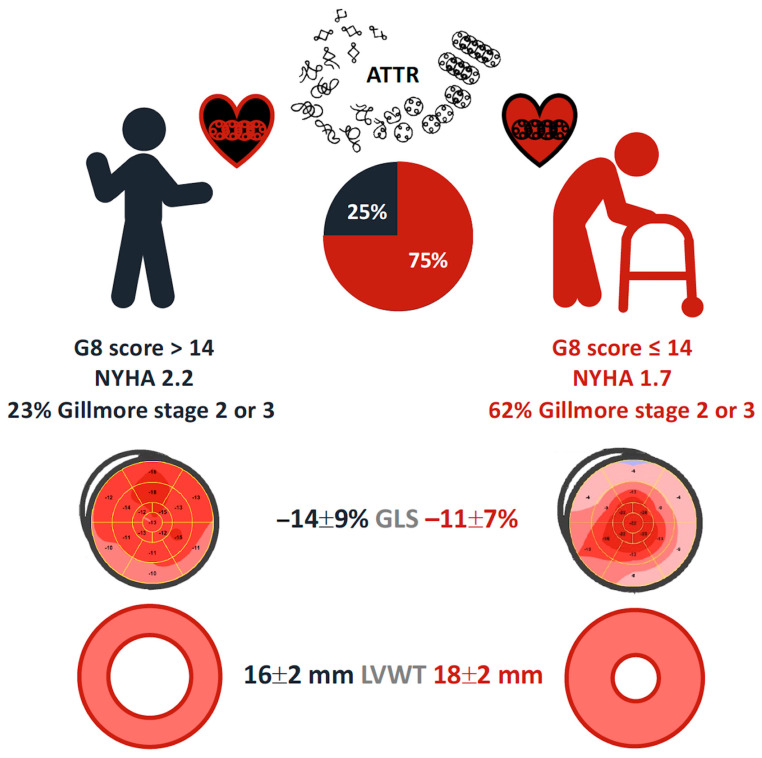
ATTR-CA patients’ profile and phenotype according to G8 score.

According to the Gilmore score, twenty-four (62%) frail patients had a Gilmore score of 2 or 3 (Figure 1), which is considered to indicate a poor prognosis, whereas the same scores were found in three (23%) non-frail patients (*p* = 0.05).

## 4. Discussion

Our study is the first to use the G8 score in ATTR-CA patients. In this study, 52 ATTR-CA patients were explored, with a mean age of 84 ± 4 years old, mainly independent according to the ADL score (mean 5.5, 96% ≥ 3) with three-quarters being frail according to the G8 score. Frail patients were more symptomatic and had more severe cardiac involvement with greater left ventricular hypertrophy, more impaired GLS, and a more severe prognosis according to the Gilmore score.

Many mechanisms could explain the link between cardiac amyloid burden and frailty. Dyspnea and fatigue are current symptoms in heart failure leading to sedentarity and the cycle of frailty. Despite its predominantly cardiac clinical expression, ATTRwt has several systemic features that may contribute to the deterioration of organ functions, such as neurological damage or presbycusis, and to the decline in intrinsic capacities, the basis of frailty. The pathophysiological association of ATTRwt with aging could explain the potentiation or even acceleration of pathological aging through frailty in the elderly.

Broussier et al. investigated overall frailty in a similar population and found that the prevalence of frailty varies according to the tests performed: 33% according to the Short Emergency Geriatric Assessment (SEGA), 50% according to the Fried criteria, and 74% according to grip strength [14]. The prevalence of frailty in heart failure phenotypes is similar, ranging from 50 to 75% depending on the tests performed and the time of the assessment (during hospitalization or ambulatory consultation) [11,33,34,35,36]. However, despite the high prevalence of frailty, patients are independent with a mean value ADL of 5.5, which could mislead cardiologists into thinking they are fit. Consequently, screening for geriatric impairments with the G8 score seems interesting and could help cardiologists identify patients requiring further CGA. 

Indeed the G8 score appears to be a time-effective tool, with a mean time to complete of about 5 min, which could be easily performed by cardiologists during outpatient consultations [21]. Further studies in ATTR patients are needed to compare this tool to CGA and to validate its future utilization in this disease.

Cardiological functional status defined by the NYHA classification and 6MWT is an important prognostic parameter and an independent factor of poor quality of life in cardiovascular disease [37]. In our study, frail patients had a higher NYHA stage than non-frail patients. However, no difference was found between the two groups regarding the walking distance in 6MWT. The absence of a difference between 6MWT in these groups shows that this functional test may not be sufficiently discriminative when identifying symptomatic older patients, unlike the NYHA classification. Indeed, most of the patients in both groups did not obtain the 6MWT predicted distance because gait speed is often altered in this population set. In younger populations, the 6MWT is an objective tool for functional status assessment and follow-up; however, in older populations, mobility is often limited by other comorbidities such as osteoarthritis, sarcopenia, etc. [38]. Among the tests for assessing functional status in heart failure, the gold standard is CPET [37]. However, in our study, less than half of the population was able to perform this examination because of reduced mobility, as previously described in an older population [39].

A causal relationship is difficult to establish on whether a patient’s cardiac functional status is the prime determinant of patient frailty or vice versa, because NYHA status does not only reflect cardiac functional status but also subjective parameters such as environmental barriers, motivation, and patients’ mood [23,39]. As such, it must be interpreted cautiously and by considering the patient’s overall health status. 

These results underline the necessity of a frailty screening and close monitoring in this population because symptomatic and frail ATTR-CA patients are at higher risk of hospitalizations, mortality, and altered quality of life, as demonstrated in patients with HFpEF [40]. 

TTE parameters showed more severe cardiac involvement with greater left ventricular hypertrophy and more impaired GLS among frail patients. These parameters, as well as elevated Nt-proBNP levels and troponin, are associated with a higher amyloid burden in this population [41,42]. However, GLS can also be affected by other concomitant pathologies in these patients such as valvular heart diseases (e.g., severe aortic stenosis), coronary artery diseases (in a subclinical state), or other comorbidities [43,44], which are all pathologies associated with frailty and confounding factors.

Finally, frail patients have a more severe prognostic score according to the Gilmore stratification of severity. Indeed, 62% were mainly classified as stage 2 or 3, compared to 23% among non-frail patients. Similarly, Broussier et al. identified an association between frailty according to the SEGA score and the Gilmore score [14]. It is well established that frailty is an important prognostic factor in heart failure, particularly in terms of mortality and hospital admissions [45]. Given the prevalence of patients requiring further CGA assessment among ATTR-CA patients, identification of frailty and early implementation of appropriate care management are essential to avoid loss of autonomy and other negative health outcomes.

### 4.1. Future Directions

This study shows the high prevalence of frailty and its association with disease stage in patients with ATTR-CA. The prognostic impact of frailty in this population needs to be confirmed by a dedicated study. These results suggest the need for systematic geriatric assessment in this population to target patients most at risk and adapt therapeutic management. Clinical trials dedicated to this particularly at-risk population could be envisaged.

### 4.2. Study Limitations

The main limitation is the small sample size and single-site setting, limiting data interpretation. Secondly, the use of the G8 score, validated in geriatric oncology, has not been validated in cardiovascular diseases and other studies are necessary to validate its use in heart failure and ATTR-CA.

## 5. Conclusions

Cardiac amyloid burden is associated with frailty according to the G8 score in ATTR-CA patients and is associated with an impaired prognostic score. Exploration of patients with ATTR-CA over 75 years old using the G8 score should be considered to implement targeted interventions.

## Figures and Tables

**Table 1:** Population classified according to frailty status.

	Overall (n = 52)	Frail (n = 39) G8 ≤ 14	Non-Frail (n = 13) G8 > 14	*p*-Value
	Mean ± SD or n (%)	Mean ± SD or n (%)	Mean ± SD or n (%)	
Age, y	84 ± 4	85 ± 4	80 ± 4	<0.001
Gender, Male	42 (81)	32 (82)	10 (77)	0.05
Weight, kg	71 ± 11	70 ± 9	75 ± 16	0.40
Height, cm	168 ± 0.1	168 ± 0.1	168 ± 0.1	0.77
Body mass index, kg/m^2^	25 ± 4	25 ± 3	26 ± 5	0.24
Geriatric assessment				
ADL score (/6)	5.5 ± 1	5.0 ± 1	6.0 ± 0.2	0.031
Medical history, n (%)				
Paroxystic atrial fibrillation	5 (10)	4 (10)	1 (8)	0.79
Permanent atrial fibrillation	22 (42)	17 (44)	5 (39)	0.75
Severe aortic stenosis	5 (10)	5 (13)	0	0.17
Presence of PM	10 (19)	7 (18)	3 (23)	0.68
NYHA stage, mean	2.1 ± 0.7	2.2 ± 0.6	1.7± 0.7	0.004
Stage I, n (%)	7 (13)	3 (7)	4 (31)	0.06
Stage II, n (%)	32 (61)	23 (60)	9 (69)	0.50
Stage III, n (%)	12 (23)	12 (30)	0 (0)	0.02
Stage IV, n (%)	1 (2)	1 (3)	0 (0)	0.50
Biology				
Hemoglobin, g/dL	13.1 ± 1.8	13.1 ± 1.9	13.0 ± 1.6	0.66
Creatinine, µmol/L	109 ± 36	112 ± 34	102± 37	0.45
High-sensitivity troponin T, μg/L	65 (42–92)	65 (45–99)	42 (28–80)	0.07
NT pro-BNP, ng/mL	2320 (1127–4083)	2912 (1193–4618)	1500 (926–2350)	0.06
Albumin, g/L	39 ± 4	39 ± 4	38 ± 4	0.78
eGFR, mL/min	55 ± 17	53 ± 15	62 ± 19	0.18
Gilmore severity score, n (%)				
Stage 1	25 (48)	15 (38)	10 (77)	0.02
Stage 2	21 (40)	19 (49)	2 (15)	0.05
Stage 3	6 (12)	5 (13)	1 (8)	0.62
Stage 2/3	27 (52)	24(62)	3 (23)	0.05
Medication				
Furosemid, n (%)	42 (80)	33 (63)	9 (17)	0.24
Mean Furosemid Posology (mg/day)	80 (40–124)	80 (40–125)	40 (40–80)	0.36
Tafamidis, n (%)	48 (92)	35 (90)	13 (100)	0.56
More than 3 medications, n (%)	47 (90)	37 (95)	10 (77)	0.09

ADL, Activities of Daily Living; NT-proBNP, N-terminal pro-B-type natriuretic peptide; eGFR, estimated glomerular filtration rate; PM, pacemaker; NYHA, New York Heart Association.

**Table 2:** Functional parameters.

	Overall	Frail G8 ≤ 14	Non Frail G8 > 14	*p*-Value
	n = 52	n = 39	n = 13	
NYHA stage, n (%)				
I	7 (13)	3 (7)	4 (31)	0.056
II	32 (62)	23 (60)	9 (69)	0.500
III	12 (23)	12 (30)	0 (0)	0.024
III/IV	13 (25)	13 (33)	0 (0)	0.023
Mean	2.1 ± 0.7	2.2 ± 0.6	1.7 ± 0.7	0.004
6MWD, m	288 ± 124	283 ± 101	305 ± 187	0.615
6MWD predict, %	52 ± 22	52 ± 18	54 ± 32	0.724
Peak VO_2_, mL/kg/min	19.5 ± 3.4	13 ± 3	16 ± 2	0.107

NYHA, New York Heart Association; 6MWD, 6 min walking distance; 6MWD % predict, 6 min walking distance percentage prediction.

**Table 3:** Echocardiographic parameters.

	Overall	Frail (n = 39) G8 ≤ 14	Non-Frail (n = 13) G8 > 14	*p*-Value
	n = 52	n = 39 (75%)	n = 13 (25%)	
Left ventricular parameters				
LV ejection fraction, %	53 ± 13	52 ± 13	57 ± 9	0.286
LV septum, mm	17 ± 2	18 ± 2	16 ± 2	0.033
LV GLS, %	12.5 ± 4.3	11.7 ± 4.3	14.9 ± 3.7	0.014
LS apex-to-base gradient	1.3 ± 0.7	1.3 ± 0.5	1.3 ± 0.8	0.178
Left ventricular diastolic function				
E velocity, cm/s	85 ± 27	82 ±26	86 ± 27	0.975
A velocity, cm/s	62 ± 25	64 ± 27	58 ± 21	0.567
Ea lateral, cm/s	6.4 ± 2.1	6.4 ± 2.1	6.7 ± 2.1	0.538
Mitral E/A ratio	1.5 ± 0.8	1.5 ± 0.8	1.5 ± 0.7	0.682
E/Ea lateral ratio	15 ± 7	15 ± 8	13 ± 6	0.547
LA volume index, mL/m^2^	45 ± 21	46 ± 22	43 ± 17	0.660
Vmax TR, cm/s	2.8 ± 0.5	2.8 ± 0.4	2.8 ± 0.5	0.995
Right ventricular systolic function				
Tricuspid annular S wave, cm/s	9.5 ± 3.0	9.4 ± 2.9	9.6 ± 3.0	0.827
Systolic PAP, mmHg	39 ± 12	39 ± 11	39 ± 12	0.847
TAPSE, mm	17 ± 5	17 ± 5	18 ± 5	0.398

<GLS, global longitudinal strain; LS, lateral strain; LV, left ventricular; LA, left atrial; PAP, pulmonary arterial pressure; TAPSE, tricuspid annular plane systolic excursion; TR, tricuspid regurgitation.

## Data Availability

Data are available upon reasonable request to the corresponding author.
